# Construction of Light-Responsive Gene Regulatory Network for Growth, Development and Secondary Metabolite Production in *Cordyceps militaris*

**DOI:** 10.3390/biology11010071

**Published:** 2022-01-04

**Authors:** Ammarin In-on, Roypim Thananusak, Marasri Ruengjitchatchawalya, Wanwipa Vongsangnak, Teeraphan Laomettachit

**Affiliations:** 1 Bioinformatics and Systems Biology Program, School of Bioresources and Technology, King Mongkut’s University of Technology Thonburi, Bangkok 10150, Thailand; Ammarin.ammarinin@mail.kmutt.ac.th (A.I.-o.); marasri.rue@kmutt.ac.th (M.R.); 2 School of Information Technology, King Mongkut’s University of Technology Thonburi, Bangkok 10150, Thailand; 3 Interdisciplinary Graduate Program in Bioscience, Faculty of Science, Kasetsart University, Bangkok 10900, Thailand; roypim.th@ku.th; 4 Biotechnology Program, School of Bioresources and Technology, King Mongkut’s University of Technology Thonburi, Bangkok 10150, Thailand; 5 Department of Zoology, Faculty of Science, Kasetsart University, Bangkok 10900, Thailand; 6 Omics Center for Agriculture, Bioresources, Food and Health, Kasetsart University (OmiKU), Bangkok 10900, Thailand; 7 Theoretical and Computational Physics (TCP) Group, Center of Excellence in Theoretical and Computational Science (TaCS-CoE), King Mongkut’s University of Technology Thonburi, Bangkok 10150, Thailand

**Keywords:** *Cordyceps militaris*, carotenoid, cordycepin, light-responsive regulation, transcription factor

## Abstract

**Simple Summary:**

*Cordyceps militaris* is an edible fungus that has been long used in traditional medicine. A large body of research has provided evidence of the medicinal properties of *C. militaris* extract and its demand has been increasing over the years. This study aims to construct and understand the light-responsive gene regulatory network of the fungi by combining the transcription factor (TF)-target gene interactions with the transcriptomic analysis of *C. militaris* under a light-programming condition. The study identified several key TFs and their gene targets that regulate growth, development and secondary metabolite production in the fungi under specific light conditions.

**Abstract:**

*Cordyceps militaris* is an edible fungus that produces many beneficial compounds, including cordycepin and carotenoid. In many fungi, growth, development and secondary metabolite production are controlled by crosstalk between light-signaling pathways and other regulatory cascades. However, little is known about the gene regulation upon light exposure in *C*. *militaris*. This study aims to construct a gene regulatory network (GRN) that responds to light in *C. militaris*. First, a genome-scale GRN was built based on transcription factor (TF)-target gene interactions predicted from the Regulatory Sequence Analysis Tools (RSAT). Then, a light-responsive GRN was extracted by integrating the transcriptomic data onto the genome-scale GRN. The light-responsive network contains 2689 genes and 6837 interactions. From the network, five TFs, Snf21 (CCM_04586), an AT-hook DNA-binding motif TF (CCM_08536), a homeobox TF (CCM_07504), a forkhead box protein L2 (CCM_02646) and a heat shock factor Hsf1 (CCM_05142), were identified as key regulators that co-regulate a large group of growth and developmental genes. The identified regulatory network and expression profiles from our analysis suggested how light may induce the growth and development of *C. militaris* into a sexual cycle. The light-mediated regulation also couples fungal development with cordycepin and carotenoid production. This study leads to an enhanced understanding of the light-responsive regulation of growth, development and secondary metabolite production in the fungi.

## 1. Introduction

*Cordyceps militaris* is an entomopathogenic fungus in the phylum Ascomycota [1]. The edible species produces many beneficial metabolites with medicinal properties including, anti-microbial, anti-HIV, anti-diabetic, anti-angiogenic, antioxidant and liver-protective [2]. One of the important metabolites produced by *C. militaris* is 3′-deoxyadenosine or cordycepin, which has been shown to inhibit tumor growth and possesses many pharmaceutical properties [2,3,4]. Co-administration of cordycepin and pentostatin, an adenosine deaminase inhibitor, has entered clinical trial phase I for chronic myelogenous leukemia treatment [5]. Another key metabolite produced by the species is carotenoid [6], a terpenoid compound with antioxidant activity [7,8]. The accumulation of carotenoids in several fungi is necessary to protect them from oxidative stress and UV irradiation. Generally, the carotenoid compounds produced from fungi include β-carotene, xanthophyll and astaxanthin [7]. The major type of carotenoid produced by *C. militaris* is a xanthophyll, called cordyxanthin, which shows better water solubility than other carotenoids [9].

The market size for *Cordyceps* sp. extract was estimated at around 470 million USD in 2018 and is forecasted to increase over the years [10]. Various environmental factors, such as pH, temperature and light, affect the growth, development and metabolite production of *C. militaris* [11,12,13]. The optimizing process for culturing *Cordyceps* sp. to increase the production of beneficial metabolites has gained much attention [14,15] based on advanced systematic analysis [16,17,18]. Previous studies have shown that light strongly affects the fruiting body formation and cordycepin and carotenoid synthesis in *C. militaris* [6,13,19], especially the carotenoid metabolism, which is increased under light-programming exposure [20].

*C. militaris* can sense light using photoreceptors. White Collar (WC) proteins and phytochromes (Phys) are two protein families which detect short-wavelength and long-wavelength lights, respectively. These photoreceptors also regulate the expression of genes that are involved in the development, stress response, secondary metabolite synthesis and circadian clock [21]. For example, White Collar-1 (WC-1), containing zinc finger DNA-binding domains, is crucial for cordycepin and carotenoid biosynthesis [12], cellular metabolism and sexual and asexual development [22].

In the filamentous fungal model organism, *Aspergillus nidulans*, under dark conditions, light receptors WC-1, WC-2 and the phytochrome Fph interact with VelvetA (VeA) inside the nucleus to form a large complex that modulates sexual development [23]. Exposure to light inhibits the translocation of VeA into the nucleus, thus leading the fungi to asexual reproduction (conidiation). In *Neurospora crassa*, the *fluffy* (*fl*) gene, which is responsible for the development of asexual spores, is activated by light via the WC complex [24]. In addition, the phytochromes control genes’ expression by sending the signal through the high-osmolarity glycerol (HOG) pathway to activate the transcription factor AtfA. This protein regulates the expression of stress response and asexual sporulating genes in fungi [25,26]. However, the light-responsive transcriptional regulation that promotes the morphological development and secondary metabolite production in *C. militaris* remains largely unknown.

Transcriptional regulatory networks of *A. nidulans* and *N. crassa* have been constructed by manually curating transcription factor (TF)-target gene interactions from literature and databases [27]. However, such information is limited for *C. militaris*. Therefore, identifying the TF-target gene interactions in the species using computational approaches provides a potential alternative. To identify target genes of TFs, the chromatin immunoprecipitation (ChIP) technique has been widely used. The method uses antibodies to precipitate TFs, which are bound to their DNA targets. Therefore, the DNA sequences in the complex can be identified. An alternative technique has been applied with microarray technology [28], called protein-binding microarray (PBM), to identify TF binding sequences. Weirauch et al. [29] analyzed data from the PBM assays of over 1000 TFs to determine DNA binding specificity represented by position frequency matrices (PFMs). The PFMs were then used to infer PFMs of other TFs from diverse eukaryotes, including *C. militaris*, based on the similarity of protein sequences [29]. To construct a gene regulatory network (GRN), the Regulatory Sequence Analysis Tools (RSAT) allows users to scan the upstream sequences of genes with the PFMs using Markov chain-based background models to identify TF binding domains [30]. The predicted TF-target binding domain interactions from RSAT are then used to infer the GRN.

This study aims to construct the GRN in *C. militaris* that responds to light. First, the collected PFMs of TFs of *C. militaris* were used to predict all possible TF binding sites in the promoter region of all genes in the *C. militaris* genome. Next, the interaction profiles between TFs and target genes were used to create a genome-scale GRN, which can be visualized as a network. Then, differentially expressed genes (DEGs) under light and dark conditions from transcriptome analysis were integrated into the network to extract a light-responsive sub-network. The light-responsive GRN revealed the transcriptional regulation of many developmental and carotenoid and cordycepin biosynthesis genes by five key TFs, enabling more understanding of the regulation of light-controlled development and secondary metabolite production in *C. militaris*.

## 2. Materials and Methods

**Figure 1:**
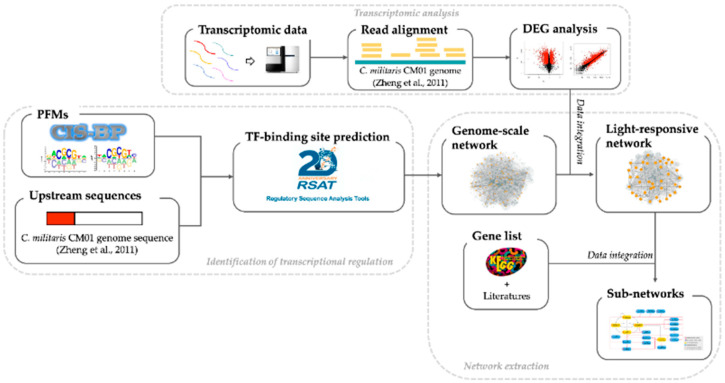
The overall workflow implemented in the current study. First, position frequency matrices (PFMs) of each TF of *C. militaris* were collected from CIS-BP. Next, Matrix-scan, a package in the Regulatory Sequence Analysis Tools (RSAT), was used to scan the upstream sequences of all *C. militaris* genes with each PFM to identify TF binding sites for each TF. The TF-target gene interactions were used to construct a genome-scale GRN. Then, differentially expressed genes (DEGs) under light and dark conditions from transcriptome analysis were integrated into the network to extract a light-responsive GRN. Finally, a list of genes involved in the development and carotenoid and cordycepin biosynthesis were used to extract light-responsive regulatory sub-networks.

The overall workflow of the present study is shown in Figure 1 and is detailed as follows.

### 2.1. Construction of the Genome-Scale GRN

The genome-scale GRN was constructed by identifying TF binding sites in the promoter region of all genes in the *C. militaris* genome. The genomic sequence of *C. militaris* was retrieved from BioProject (PRJNA41129) [31]. The upstream sequences from −1 to −1000 bp were extracted from 9651 genes. All 488 PFMs of 83 TFs of *C. militaris* were retrieved from the CIS-BP database version 2.0 (cisbp.ccbr.utoronto.ca, accessed on 6 December 2019) [29]. Matrix-scan, a package in the Regulatory Sequence Analysis Tools (RSAT) [30], was used to scan the upstream sequences with each PFM to identify TF binding sites for each TF in the genome. A higher-order (*m* = 2) Markov model was used as a background model with pseudo-frequencies = 0.001. Only predictions with a *p*-value ≤ 10^−5^ were selected to reduce the false positives. The predicted interactions between TFs and their target genes were constructed as a genome-scale GRN in Cytoscape version 3.7.2 (https://cytoscape.org, accessed on 22 October 2019) [32], where nodes represent TFs and target genes and edges represent TF-target gene interactions.

### 2.2. Differentially Expressed Gene (DEG) Analysis and the Light-Responsive GRN

RNA sequencing reads of *C. militaris* strain TBRC6039 in light condition (BioProject: PRJNA579732, BioSample SAMN13118310) and dark condition (BioProject: PRJNA416937, BioSample SAMN07969453) were retrieved from previous studies [20,33]. Reads were filtered by the quality control at the Phred-score ≥30, yielding qualified reads of 88.68% and 94.46% in light and dark condition samples, respectively. The mapping process was performed using STAR two-pass-mode [34] version 2.7.3a. The reference genome of *C. militaris* was retrieved from BioProject (PRJNA41129) [31]. Uniquely mapped reads were more than 80%, while only 2.2% percent of the reads were unmapped in both light and dark conditions (Table S1 in Supplementary File S1). Non-uniquely mapped reads (18.4% and 16.1% of the total reads in light and dark conditions, respectively) and reads mapped with less than five reads (<0.005% of the total reads in both conditions) were removed. Finally, uniquely mapped reads with ≥5 mapped reads were counted by STAR using quantMode GeneCounts [34]. Read counts per gene in light and dark conditions were normalized using the trimmed mean of M values (TMM) method [35]. The normalization method is suitable for normalizing raw read counts between inter- and intra-samples before differentially expressed gene (DEG) analysis by NOISeq-sim [35]. The NOISeq-sim tool simulates the technical replication from the sequencing depth of input data [36]. Parameters of the simulation were set as follows: the percentage of the total number of reads (*pnr*) = 0.2, the number of simulated samples (*nss*) = 10 and variability in sample total reads of simulated samples (*v*) = 0.02. These parameters were set similarly to a previous analysis [20], except the value of *nss*, which was strictly set to reduce the false positives. NOISeq-sim calculates the probability of differential expression (*q*), which was set to be *q* ≥ 0.9 for DEGs in this study. The list of DEGs was then used to extract nodes and all connected edges among the nodes from the genome-scale GRN to construct the light-responsive GRN.

### 2.3. Identification of Key Regulator Genes

Key TFs that play an essential role in the global regulation of the network were identified based primarily on the outdegree of the nodes in the light-responsive GRN. Other node measures were also used for the analysis, including the normalized betweenness centrality score by cytoHubba, a plugin in Cytoscape [37] and the motif-based score calculated using an in-house script.

The betweenness centrality score of node *n* is defined as

$$B\left(n\right)=\sum_{i\neq j\neq n\in C}\frac{\sigma_{ij}\left(n\right)}{\sigma_{ij}}\;,$$

where $\sigma_{ij}$ is the number of shortest paths between node *i* and *j* and $\sigma_{ij}$(*n*) is the number of shortest paths between node *i* and *j* that walk through node *n*. C represents the collection of all nodes in the network. The betweenness score of each node can be normalized by

$$\mathrm{Normalized}\;\mathrm{betweenness}\;\mathrm{score}\;\mathrm{of}\;\mathrm{node}\;n=\frac{B\left(n\right)-\min\left(B\right)}{\max\left(B\right)-\min\left(B\right)}\;,$$

where *B*(*n*) is the betweenness score of node *n*, max(*B*) and min(*B*) are the maximum and minimum betweenness scores of all nodes in the network.

The motif-based scores were calculated by counting the number of times a node participates in a feed-forward loop (FFL) motif as the master regulator, intermediate regulator, or target [38].

### 2.4. The Gene Regulatory Sub-Networks of Growth, Development, Carotenoid and Cordycepin Biosynthesis Pathways under Light Response

A list of genes involved in morphological development, carotenoid and cordycepin biosynthesis of model fungi was obtained from previous studies [12,39,40,41,42,43,44] and the Kyoto Encyclopedia of Genes and Genomes (KEGG) database (https://www.genome.jp/kegg/, accessed on 6 December 2019) [45]. The genes from *C. militaris* were identified based on homology analysis, which was done by bi-directional BLASTp with criteria evalue ≤10^−10^, percent identity ≥60% and percent coverage ≥40%. The development and biosynthesis subnetworks were created by extracting the light-responsive GRN with DEGs from the list and their parent TFs in the network.

## 3. Results and Discussion

### 3.1. The Genome-Scale GRN

The prediction results from RSAT’s matrix-scan indicated 86,172 TF binding sites from 8584 genes. From a total of 83 TFs, 71 TFs were identified to regulate genes, resulting in 31,703 interactions between TFs and target genes (Figure S1 in Supplementary File S2). The average outdegree of 71 TFs in the network was approximately 451, with a median of 161, indicating that some TFs regulate a large number of nodes in the network. All TF-target gene interactions in the genome-scale GRN are listed in Supplementary File S3.

### 3.2. The Light-Responsive GRN

**Figure 2:**
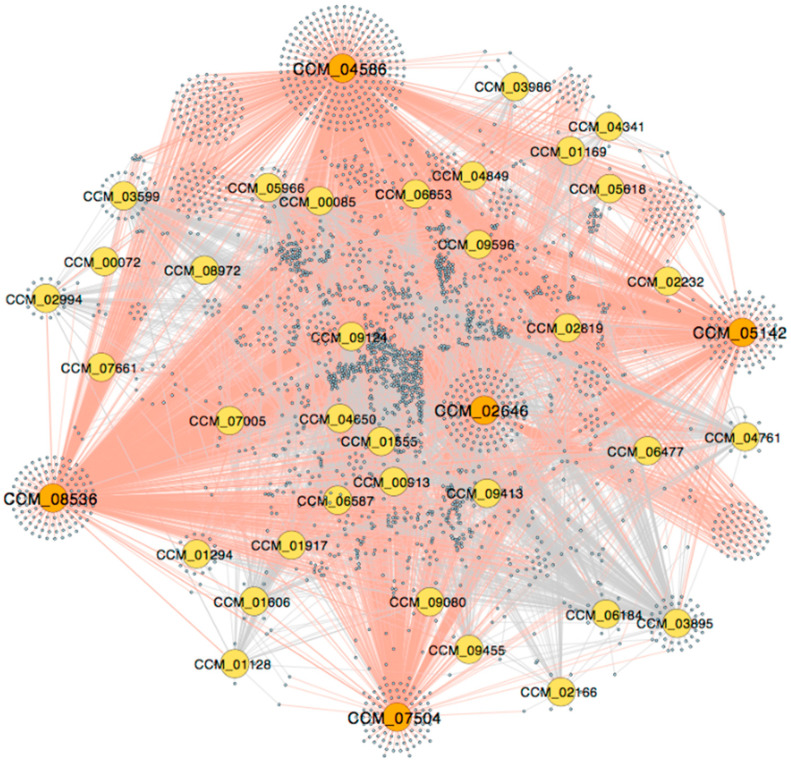
The light-responsive GRN. Five large orange nodes represent the key TFs that directly regulate almost over 90% of genes in the network. The direct regulation by the key TFs is labeled by red edges. Other TFs (non-key regulators) are shown as yellow nodes and other target genes are represented by small blue nodes.

To construct the light-responsive GRN, DEGs upon light stimulation were mapped into the genome-scale GRN and the DEG nodes along with edges among them were extracted. DEG nodes that did not connect to any other DEG nodes were removed. The extracted light-responsive GRN is visualized in Figure 2. Originally, the number of DEGs before mapping was 3256 (34.72% of the expressed genes), distributing into 1520 and 1736 up-regulated and down-regulated genes, respectively. After mapping and extracting, the light-responsive network contains 2689 genes (31.32% of nodes in the genome-scale GRN) and 6837 interactions (21.57% of interactions in the genome-scale GRN). There are 39 TFs out of 71 TFs (54.94%) in the network. TF-target gene interactions in the light-responsive GRN are listed in Supplementary File S4.

**Figure 3:**
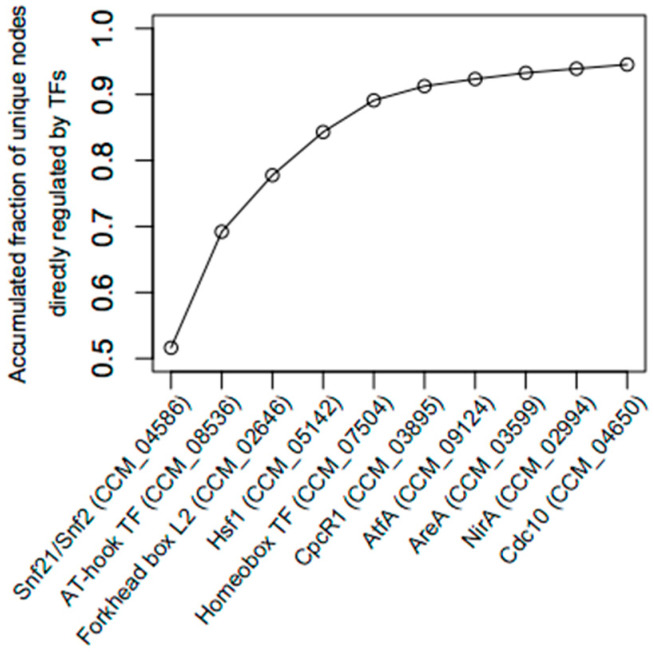
The accumulated fraction of unique genes directly regulated by TFs. The fraction rises to ~0.9 when the first five TFs are included. Here, they are assigned as key TFs.

Table 1 displays several node measures of 10 TFs in the light-responsive GRN. The 10 TFs are ranked in an order that achieves the maximum accumulated number of unique nodes directly regulated by the TFs (Figure 3). For example, the first TF, Snf21, an Snf2-family helicase (CCM_04586), has the highest outdegree in the light-responsive GRN (1388 targets, approximately 52% of all nodes). The second TF is an AT-hook DNA-binding motif TF (CCM_08536) with an outdegree of 1044 target genes. Together, the AT-hook DNA-binding motif TF and Snf21 regulate 1861 unique target genes, approximately 70% of the total nodes. As shown in Figure 3, the top five TFs regulate about 90% (2396 genes) of the total nodes in the light-responsive GRN. Hereby, this study assigned these five TFs as the key regulators of the light-responsive GRN.

**Figure 4:**
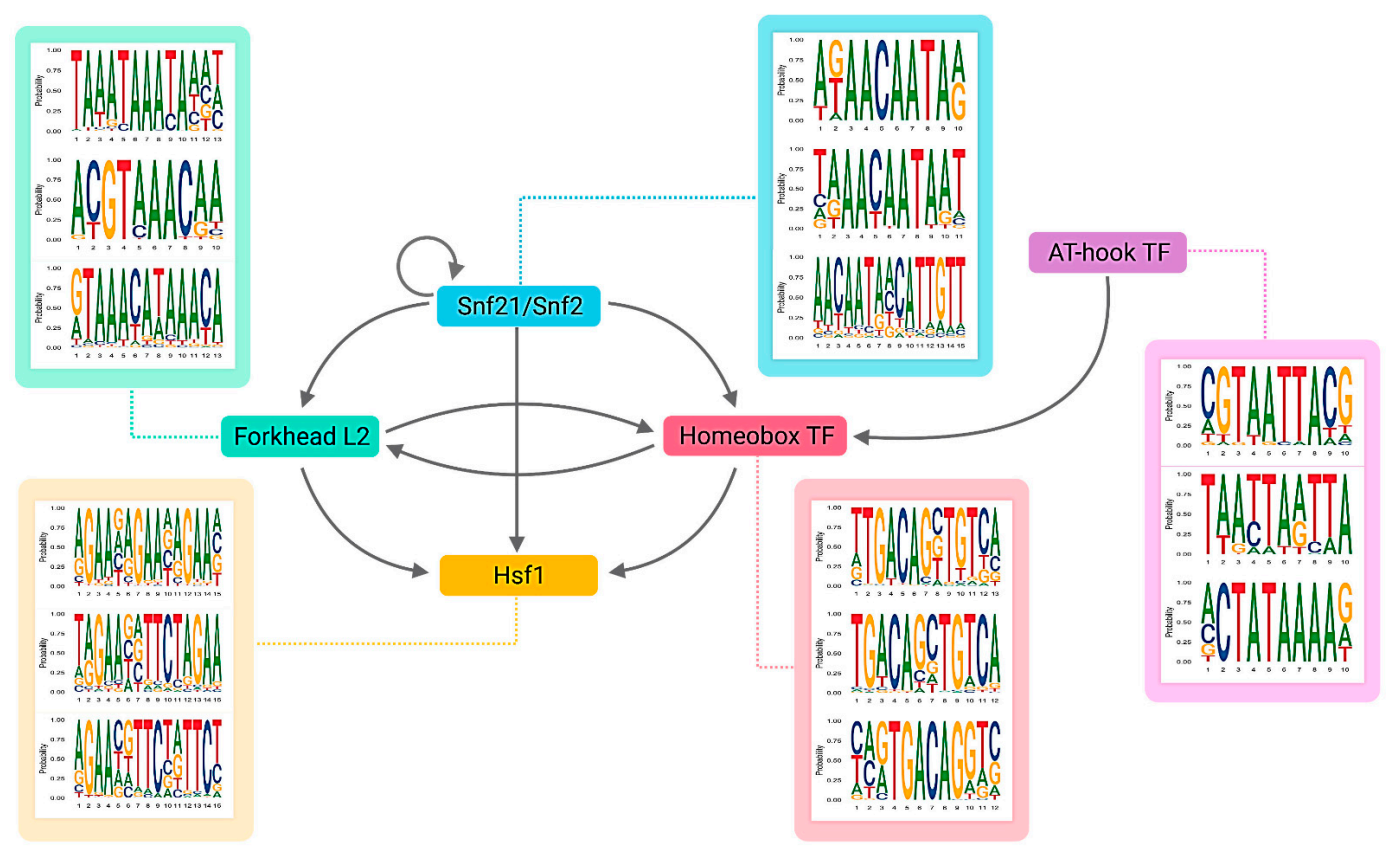
The regulatory sub-network of five key TFs including Snf21 (CCM_04586), a forkhead box protein L2 (CCM_02646), a homeobox TF (CCM_07504), Hsf1 (CCM_05142) and an AT-hook DNA-binding TF (CCM_08536). The figure also shows the examples of the DNA-binding domains for each TF predicted by RSAT.

The five key TFs belong to general TF families identified in fungi [46]. In addition, the network reveals that the five key TFs regulate one another by forming several network motifs, as shown in Figure 4. Snf21 regulates a homeobox TF (CCM_07504), a forkhead box protein L2 (CCM_02646) and Heat shock factor 1 (Hsf1) (CCM_05142) in multiple feed-forward loops (FFLs). The AT-hook DNA-binding motif TF (CCM_08536) co-regulates the homeobox TF (CCM_07504) with Snf21. Finally, the homeobox TF and the forkhead box protein L2 engage in a feedback loop.

Snf21 participates in 1407 feed-forward loops in the light-responsive network, where Snf21 is always the master regulator of the motifs (Table 1), implying a regulator role of Snf21 in the global control of the genes in *C. militaris* under light conditions. Further analysis shows that Snf21/Snf2 is an ortholog of SWI2 in baking yeast. Baking yeast’s SWI2 is involved in the global transcriptional activation of many genes [47]. The protein also displays a conserved function for activating the transcription by controlling the chromatin structure [48,49]. In *Schizosaccharomyces pombe*, Snf2 is important to mitosis and vegetative growth [50]. TFs in the Snf2 family in *Arabidopsis* spp. and higher plants are involved in abiotic and oxidative stress responses [51,52,53].

Heat shock factor 1 (Hsf1) is another interesting TF in the light-responsive network. The TF has the highest betweenness score (a normalized betweenness score of 1.0), indicating it as the most central node that participates in the regulatory flow of other nodes in the network [38]. Hsf1 is activated by light and relates to fatty acid metabolism and circadian rhythm in eukaryotic organisms [54]. In yeast, Hsf1 is also required for growth at a normal temperature [55,56]. In *C. militaris*, besides light, heat is also a factor affecting cordycepin and carotenoid production [19].

### 3.3. Growth and Developmental Regulation under Light Exposure

**Figure 5:**
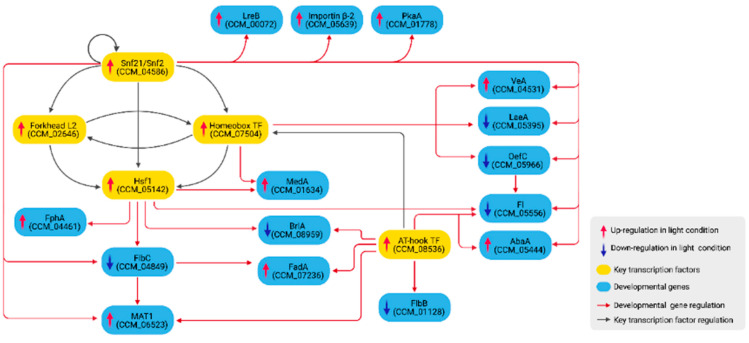
The regulatory sub-network of growth and developmental genes controlled by the key TFs.

Growth and developmental genes in *C. militaris* were identified based on homology analysis against known genes in other model fungi (Table 2 and Supplementary File S5). To reveal how the genes are regulated, only DEGs in Table 2 and all upstream nodes from the light-responsive network were extracted. Figure 5 shows 15 growth and developmental genes that are included in the sub-network and are direct targets of the five key TFs.

*lreA* (CCM_04514), *lreB* (CCM_00072), *fphA* (CCM_04461), *veA* (CCM_04531) and *laeA* (CCM_05395), encoding proteins that form the light-regulatory VeA/FphA/LreA/LreB complex [57] and the VeA/LaeA complex [42], were all differentially expressed upon light exposure. The complexes translocate into the nucleus and regulate asexual/sexual development balance [23]. VeA is a positive regulator of sexual fruiting body formation [58]. A *veA* mutation in *A. nidulans* reduced the development of sexual organs [23]. VeA has also been reported to be involved in the metabolism of nitrate and secondary metabolite in *F. oxysporum* [59].

Blue-light receptors LreA and LreB are activators of sexual development [57]. In contrast, red-light-mediated FphA inhibits fruiting body development while promoting conidiation in *A. nidulans* [42,60]. However, in *C. militaris*, light is essential for normal fruiting body formation [12]. LaeA is a negative regulator of sexual development [61] and VeA was shown to be the repressor of LaeA [59].

In the network (Figure 5), Snf21 up-regulates *lreB*. Snf21 also forms an FFL with the homeobox TF (CCM_07504) to up-regulate and down-regulate *veA* and *laeA*, respectively. *lreA* was down-regulated in the transcriptomic data, but it was not included in Figure 5 because the analysis found no regulatory links between *lreA* and other genes. In addition, Hsf1 up-regulates the expression of *fphA*. Therefore, the analysis reveals Snf21, the homeobox TF (CCM_07504) and Hsf1 to be the main regulators of the VeA pathway in *C. militaris*. Evidence has supported that the homeobox TF (CCM_07504) was related to fruiting body formation and the mycelial growth of *C. militaris* [62]. Additionally, Snf21 up-regulates CCM_05639, importin β-2 subunit, which may involve in the translocation of the VeA complexes into the nucleus [63].

Snf21 and the homeobox TF (CCM_07504) also form an FFL to regulate *oefC*. The OefC protein promotes asexual development in *A. nidulans* [64]. The current study also identified *fl* (CCM_05556) as a target gene of OefC. In *N. crassa*, the *fl* gene encodes Fluffy, a transcriptional activator of several genes regulating conidiation [43]. In the network, both *oefC* and *fl* were down-regulated under light exposure. Consistent with a previous study, an *oefC* homolog in *C. guangdongensis* was also shown to down-regulate under light treatment [22].

In addition, Snf21 and Hsf1 down-regulate CCM_04849, which is identified as an *flbC* ortholog. Consistent with a previous study in *C. militaris*, it was found that CCM_04849 was regulated by light [12]. In *A. nidulans*, FlbC is required for proper conidiation initiation and the deletion of the gene delayed the conidiation process while enhancing fruiting body formation [65]. FlbC, along with Snf21 and the AT-hook DNA-binding motif TF (CCM_08536), regulates the expression of *mat1* (CCM_06523), a sexual developmental gene [66]. FlbC and the AT-hook DNA-binding motif TF (CCM_08536) also regulate the expression of *fadA*. FadA is a signaling protein whose one of the substrate targets is PkaA, a regulator of sexual development [67]. In the network, the *pkaA* gene is up-regulated by Snf21.

Further, Hsf1 and the AT-hook DNA-binding motif TF (CCM_08536) down-regulate *brlA* (CCM_08959), an asexual developmental gene. Hsf1 also forms an FFL with the homeobox TF (CCM_07504) to up-regulate *medA*. BrlA was shown to transcriptionally regulate MedA and AbaA [44]. In the network, *abaA* is up-regulated by Snf21 and the AT-hook DNA-binding motif TF (CCM_08536). MedA and AbaA promote asexual growth [68] but also function in sexual development [44,69]. A *medA* mutant formed incomplete Hülle cells and did not produce cleistothecia [69]. Another asexual development-promoting gene identified in the network is *flbB*, which is down-regulated by the AT-hook DNA-binding motif TF (CCM_08536).

Taken together, the overall regulation in Figure 5 and expression profiles from the transcriptomic analysis suggest the transcriptional regulation of light that induces the growth and development of *C. militaris* into a sexual cycle by inactivating asexual developmental genes (*flbB*, *flbC*, *oefC*, *brlA* and *fl*) and activating sexual developmental genes (*veA*, *fadA*, *pkaA* and *mat1*).

It is important to note that the network in Figure 5 is far from complete. Many possible TFs were not assigned with the PFMs in CIS-BP and this study could not predict their regulatory links to target genes. For example, in *A. nidulans*, BrlA and AbaA are TFs that regulate one another [44,69]. These relationships were not identified in the present work.

The identified network in Figure 5 possesses several cascaded feed-forward loops (FFLs) made by regulatory interactions between the key TFs and other regulators. FFLs have been shown to play important roles in development, including temporal activation of genes or protection against transient loss of signals [70,71,72]. The current study identified Hsf1 as a target in two inter-connected FFLs, composed of Snf21-the homeobox TF-Hsf1 and Snf21-forkhead box L2-Hsf1. Based on the most frequent binding domain identified by RSAT (Figure 4), Snf21 binds to an AACAAT sequence, while the homeobox TF binds to TGACA, one of the conserved homeodomains reported in previous studies [73]. In addition, the forkhead box L2 recognizes the conserved domain GTAAACAA, similar to the forkhead box L1 [74]. Snf21 and Hsf1 form an FFL to regulate FlbC (CCM_04849). RSAT prediction identified (A)ACAAT and GAAnnTTCnnGAA domains in the promoter region of *flbC*. The (A)ACAAT is a TF-binding domain of Snf21, while GAAnnTTCnnGAA represents the heat shock element (HSE), a conserved binding domain of HSF with a repeated nGAAn or/and nTTCn sequence [75,76]. Furthermore, Snf21 and FlbC form another FFL to regulate *mat1*. Snf21 also forms several FFLs with the homeobox TF to regulate *veA*, *laeA* and *oefC*. We propose that these connected FFLs made of the key TFs and other regulators play important roles in the light-responsive regulation of growth and development in the fungi.

### 3.4. Light-Mediated Control of Secondary Metabolism

Light is an important factor affecting secondary metabolism in *C. militaris*. A study by Yang et al. [12] revealed that the fungi without the blue-light receptor gene (*wc-1*) showed a significant reduction in both cordycepin and carotenoid. Other evidence also suggested that the carotenoid content of *C. militaris* is enhanced by light [6,13], although light stress during the late growth stage may reduce carotenoid production. [19].

**Figure 6:**
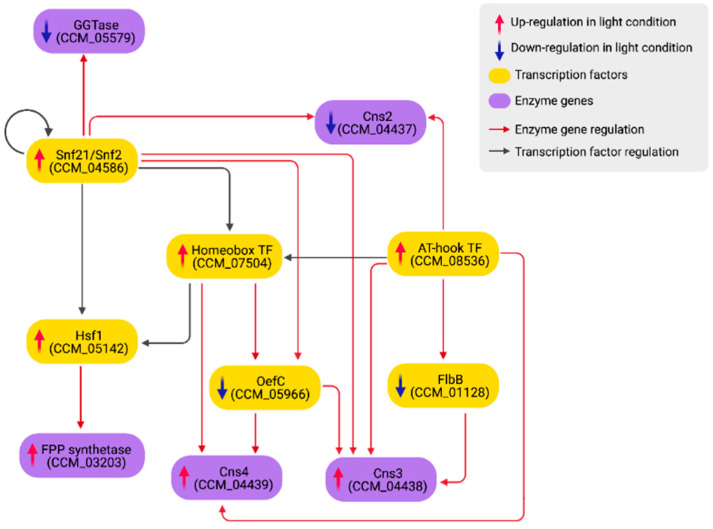
The regulatory sub-network of cordycepin/pentostatin (Cns2, Cns3 and Cns4) and carotenoid (GGTase and FPP synthetase) biosynthesis controlled by the key TFs.

The carotenoid content measured in the culture from which the transcriptome in this study was extracted increased significantly in light compared to dark conditions [20]. Similar to a previous study [12], five orthologous genes of *N. crassa* carotenoid biosynthetic genes were not DEGs in this study (CCM_03697, CCM_03059, CCM_06355 geranylgeranyl diphosphate synthases, CCM_06728 carotenoid oxygenase 2 and CCM_09155 aldehyde dehydrogenase). Rather, it was identified that CCM_03203 FPP synthetase was up-regulated, while CCM_05579 geranylgeranyltransferase (GGTase) beta subunit was down-regulated. FPP synthetase catalyzes reactions that yield farnesyl pyrophosphate (FPP), a precursor of steroids and geranylgeranyl diphosphate (GGPP), a precursor of carotenoids [77]. GGTase is a CAAX protein prenyltransferase, utilizing GGPP as a substrate to perform the prenylation process on another CAAX-type protein, such as Ras1 [78,79]. Thus, the up-regulation of FPP synthetase and the down-regulation of GGTase may explain the increase of carotenoid production under light conditions [20]. In the light-responsive GRN, Hsf1 up-regulates the FPP synthetase gene (CCM_03203), while Snf21 down-regulates the GGTase gene (CCM_05579) (Figure 6).

Xia et al. [41] previously reported four enzymes (Cns1-Cns4) regulating the cordycepin production in *C. militaris*. Cns1/Cns2 is responsible for cordycepin biosynthesis. Cns3 and Cns4 are responsible for pentostatin (PTN) production and exportation, respectively. PTN is an adenosine deaminase inhibitor, which helps protect cordycepin from deamination. In the current study, *cns1* (CCM_04436) was not a DEG, *cns2* (CCM_04437) was down-regulated, while *cns3* (CCM_04438) and *cns4* (CCM_04439) were up-regulated. Thus, it is predicted that light may induce the production and exportation of PTN. As shown in Figure 6, *cns2* is directly suppressed by Snf21 and the AT-hook DNA-binding motif TF (CCM_08536). *cns3* is regulated by four TFs, including Snf21, the AT-hook DNA-binding motif TF (CCM_08536), Zn(II)_2_Cys_6_ TF OefC (CCM_05966) and FlbB (CCM_01128). OefC and FlbB are also regulators of asexual development [64]. Under light stimulation, both genes were down-regulated, allowing *cns3* to be activated. *oefC* is suppressed by Snf21 and the homeobox TF (CCM_07504), while *flbB* is down-regulated by the AT-hook DNA-binding motif TF (CCM_08536) in the network (Figure 6). OefC, along with the AT-hook DNA-binding motif TF and the homeobox TF, participates in the regulation of *cns4*. Taken together, we propose that PTN production and an asexual cycle may be inversely coupled, where light and dark conditions promote the former and the latter, respectively.

## 4. Conclusions

This study combined the TF-target gene interactions predicted from RSAT with the transcriptomic analysis of *C. militaris* under a light-programming condition to construct the GRN that responds to light. In addition, the integration of the two information filters out false-positive predictions from both TF-target interaction and DEG analysis.

The current study identified five TFs (Snf21 (CCM_04586), an AT-hook DNA-binding motif TF (CCM_08536), a forkhead box protein L2 (CCM_02646), a homeobox TF (CCM_07504) and Hsf1 (CCM_05142)) that play key roles in the light-responsive regulation. The five TFs regulate almost over 90% of the total nodes in the light-responsive network, including genes involved in the growth and developmental programming and secondary metabolite production.

**Figure 7:**
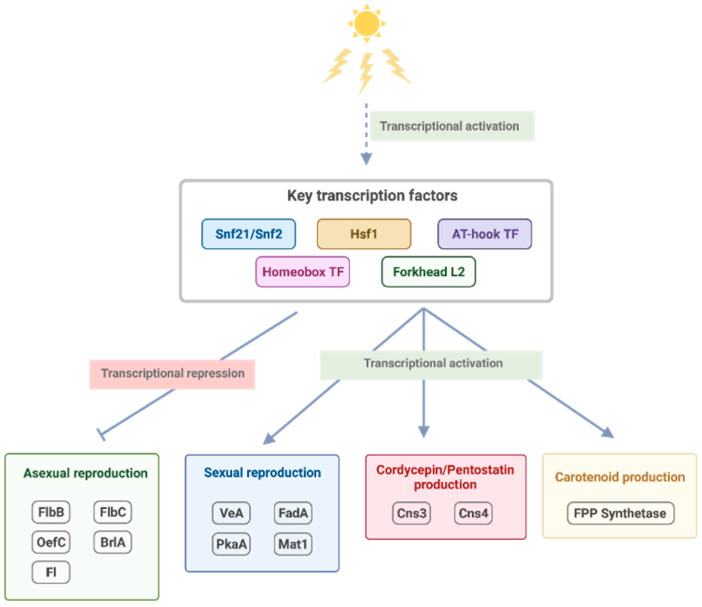
The putative light-mediated regulatory events that promote sexual growth and secondary metabolite production identified from the current study.

Additionally, this study identified a regulatory cascade made of several FFLs that co-regulate a large group of growth and developmental genes (Figure 5). The analysis suggested that upon light stimulation, these connected FFLs regulate a decision switch between asexual and sexual growth by turning off several asexual developmental and conidiation-related genes, including *flbB*, *flbC*, *oefC*, *brlA* and *fl* while turning on genes involved in sexual development such as *veA*, *fadA*, *pkaA* and *mat1*. The light-mediated regulation is also coupled with genes involved in the biosynthesis of carotenoid and cordycepin/PTN (Figure 6). Hsf1 up-regulates FPP synthetase (CCM_03203), while Snf21 down-regulates GGTase (CCM_05579), coupling fungal development with carotenoid production. Light also down-regulates OefC and FlbB, disabling asexual development while mediating the production of PTN, which may indirectly stabilize cordycepin from deamination. Figure 7 summarizes regulatory events that promote sexual growth and secondary metabolite production identified from the current study.

It should be noted that the other environment-influenced genes in our network still await further investigation. For example, several heat shock proteins were differentially expressed under light treatment, including CCM_06821, CCM_07508 and CCM_04804, which were also involved in the oxidative and osmotic stress responses in *C. militaris* in another study [80]. Additionally, two circadian clocks (CCM_01014; Frq and CCM_00908; Per) were down-regulated by the AT-hook DNA-binding motif TF (CCM_08536) and the homeobox TF (CCM_07504), respectively, under light exposure.

In summary, the construction of the light-responsive GRN reveals complex regulation between key TFs and target genes that leads to more understanding in light-regulating growth, development and secondary metabolite production in *C*. *militaris*. Nevertheless, further attempts to integrate the GRN with protein signaling and metabolic networks are needed to provide more complete descriptions of light-responsive processes in the fungi. This knowledge will enable more efficient approaches for the cultivation and beneficial metabolite production.

## Figures and Tables

**Table 1:** Node measures of 10 TFs in the light-responsive GRN.

Gene ID	TFs	Outdegree	Master	Intermediate	Target	Betweenness	DEG
CCM_04586	Snf21/Snf2 family helicase (putative)	1388	1407	0	0	0.00	up
CCM_08536	AT-hook DNA-binding motif TF	1044	460	9	0	0.06	up
CCM_02646	Forkhead box protein L2	787	497	561	1	0.25	up
CCM_05142	Heat shock factor Hsf1 (putative)	566	110	521	5	1.00	up
CCM_07504	Homeobox TF (putative)	507	285	568	1	0.49	up
CCM_03895	Cephalosporin C regulator 1 (CpcR1)	259	26	177	1	0.33	up
CCM_09124	bZIP TF (AtfA) (putative)	159	2	6	0	0.15	down
CCM_03599	Nitrogen regulatory protein AreA	152	0	11	0	0.27	up
CCM_02994	Nitrogen assimilation TF NirA	70	20	52	1	0.49	up
CCM_04650	Start control protein Cdc10	72	0	55	3	0.10	up

Note: The 10 TFs are ranked in an order that achieves the maximum accumulated number of unique nodes directly regulated by the TFs (see Figure 3). Outdegree represents the number of nodes directly regulated by the TF; Master means the number of times the TF acts as the master regulator of an FFL; Intermediate means the number of times the TF acts as the intermediate regulator of an FFL; Target means the number of times the node acts as the target of an FFL; Betweenness means normalized betweenness centrality score.

**Table 2:** List of putative genes, protein functions involved in the growth and developmental process and their orthologs. The homology analysis results are available in Supplementary File S5.

Gene ID	Description	Gene Symbol	DEG	Ortholog
CCM_00072	Cutinase palindrome-binding protein	*lreB*	Up	*lreB*, *white collar*-2 (*Neurospora crassa* OR74A), Cutinase palindrome-binding protein (*Cordyceps fumosorosea* ARSEF 2679)
CCM_00560	Sexual development transcription factor NsdD	*nsdD*	-	*nsdD* (*Cordyceps javanica, Beauveria brongniartii* RCEF 3172)
CCM_01106	C_2_H_2_ transcription factor (Egr2) putative	*egr2*	-	Conidial separation-1 (*Beauveria bassiana* ARSEF_2860), Transcriptional repressor (*C. javanica*)
CCM_01128	Hypothetical protein	*flbB*	Down	BZIP-type transcription factor (*C. fumosorosea* ARSEF 2679, *B. bassiana* ARSEF 2860)
CCM_01444	Transcription factor SteA	*steA*	-	*brlA*, *steA* (*Akanthomyces lecanii* RCEF 1005, *C. fumosorosea* ARSEF 2679)
CCM_01634	Transcriptional regulator Medusa	*medA*	Up	Transcriptional regulator Medusa, *medA* (*A. lecanii* RCEF 1005, *C. javanica*)
CCM_01778	cAMP-dependent protein kinase type 3	*pkaA*	Up	*pkaA* cAMP dependent protein kinase A catalytic subunit (*A. lecanii* RCEF 1005, *C. javanica*)
CCM_04022	DNA-binding protein creA	*creA*	-	*creA* (*C. javanica*), Carbon catabolite repressor (*A. lecanii* RCEF 1005)
CCM_04461	Sensor histidine kinase/response regulator putative	*fphA*	Up	Sensor histidine kinase/response regulator (*C. fumosorosea* ARSEF 2679, *C. javanica*)
CCM_04514	GATA transcription factor LreA	*lreA*	Down	Vivid PAS protein VVD (*C. javanica*), *lreA* (*C. fumosorosea* ARSEF 2679)
CCM_04531	Sexual development activator VeA	*veA*	Up	*veA* (*B. brongniartii* RCEF 3172, *C. javanica*)
CCM_04849	Uncharacterized protein	*flbC*	Down	C_2_H_2_ zinc finger protein *flbC* (*C. javanica*, *A. lecanii* RCEF 1005)
CCM_05395	Methyltransferase LaeA	*laeA*	Down	Methyltransferase *laeA* (*C. fumosorosea* ARSEF 267, *C. javanica*)
CCM_05444	Transcription factor AbaA (Conidiophore development regulator)	*abaA*	Up	*abaA* (*C. fumosorosea* ARSEF 2679, *B. brongniartii* RCEF 3172)
CCM_05556	Fungal transcriptional regulatory protein	*fl*	Down	Fluffy (*B. brongniartii* RCEF 3172)
CCM_05639	Importin β-2 subunit	*kapB*	Up	Importin beta-2 subunit (*B. bassiana* ARSEF 2860, *C. javanica*)
CCM_05697	Glutamine synthetase	*fluG*	-	Glutamine synthetase (*C. fumosorosea* ARSEF 2679, *C. javanica*)
CCM_05966	OefC protein	*oefC*	Down	*oefC* (*B. bassiana* ARSEF 2860, *C. javanica*)
CCM_06523	Mating-type protein MAT 1-1-1	*mat1*	Up	Mating-type protein MAT1-1-1 (*B. bassiana* ARSEF 2860, *A. lecanii* RCEF 1005)
CCM_07203	Developmental regulator FlbA	*flbA*	-	*flbA* (*C. fumosorosea* ARSEF 2679, *C. javanica*)
CCM_07236	Guanine nucleotide-binding protein alpha subunit	*fadA*	Up	*fadA* (*B. bassiana* ARSEF 2860, *Aspergillus fumigatus Af293*)
CCM_08391	Hypothetical protein	*wetA*	-	Developmental regulatory protein WetA (*Fusarium phyllophilum*)
CCM_08959	Hypothetical protein	*brlA*	Down	*brlA* (*B. bassiana* ARSEF_2860, *C. fumosorosea* ARSEF 2679)
CCM_09566	DNA-binding protein eta putative	*flbD*	-	*flbD*, Myb-like DNA-binding domain (*Madurella mycetomatis*)

## Data Availability

RNA sequencing reads of *C. militaris* in light and dark conditions were retrieved from previous studies [20,33] with BioProject PRJNA579732, BioSample SAMN13118310 and BioProject PRJNA416937, BioSample SAMN07969453, respectively. The genomic sequence of *C. militaris* was retrieved from BioProject PRJNA41129 [31].

## Supplementary Materials

The following are available online at https://www.mdpi.com/article/10.3390/biology11010071/s1 Supplementary File S1: Read mapping result, Supplementary File S2: Genome-scale GRN figure, Supplementary File S3: Genome-scale GRN table, Supplementary File S4: Light-responsive GRN table, Supplementary File S5: Protein homology analysis results.
