# Supplementary material for: Construction of Light-Responsive Gene Regulatory Network for Growth, Development and Secondary Metabolite Production in Cordyceps militaris
Source: Biology (Basel). 2022 Jan 4;11(1):71. doi: 10.3390/biology11010071 (PMC8773263; doi:10.3390/biology11010071)
Supplement: Supplementary file 1 [file biology-11-00071-s001.zip › biology-1523686-supplementary/Supplementary file S1.pdf]

Table S1. The summary results from STAR's read mapping to the reference genome (*C. militaris* CM01). Only uniquely mapped reads are used further in DEG analysis.

Type of mapping	Light condition	Dark condition
Uniquely mapped (read)	19,904,584 (81.6%)	18,937,867 (83.9%)
Unmapped read (read)	544,188 (2.2%)	498,398 (2.2%)
Multi mapping (read)	68,962 (0.3%)	53,431 (0.2%)
Ambiguous (read) *	26,576 (0.1%)	17,689 (0.1%)
No feature (read) **	3,862,619 (15.8%)	3,067,318 (13.6%)
Total reads	24,406,979	22,574,703

* Ambiguous reads are the reads that are non-uniquely mappable to at least two genes in the reference genome.

** No feature reads refer to the reads that are not aligned perfectly to the reference sequence.
