# Supplementary material for: Construction of Light-Responsive Gene Regulatory Network for Growth, Development and Secondary Metabolite Production in Cordyceps militaris
Source: Biology (Basel). 2022 Jan 4;11(1):71. doi: 10.3390/biology11010071 (PMC8773263; doi:10.3390/biology11010071)
Supplement: Supplementary file 1 [file biology-11-00071-s001.zip › biology-1523686-supplementary/Supplementary file S2.pdf]

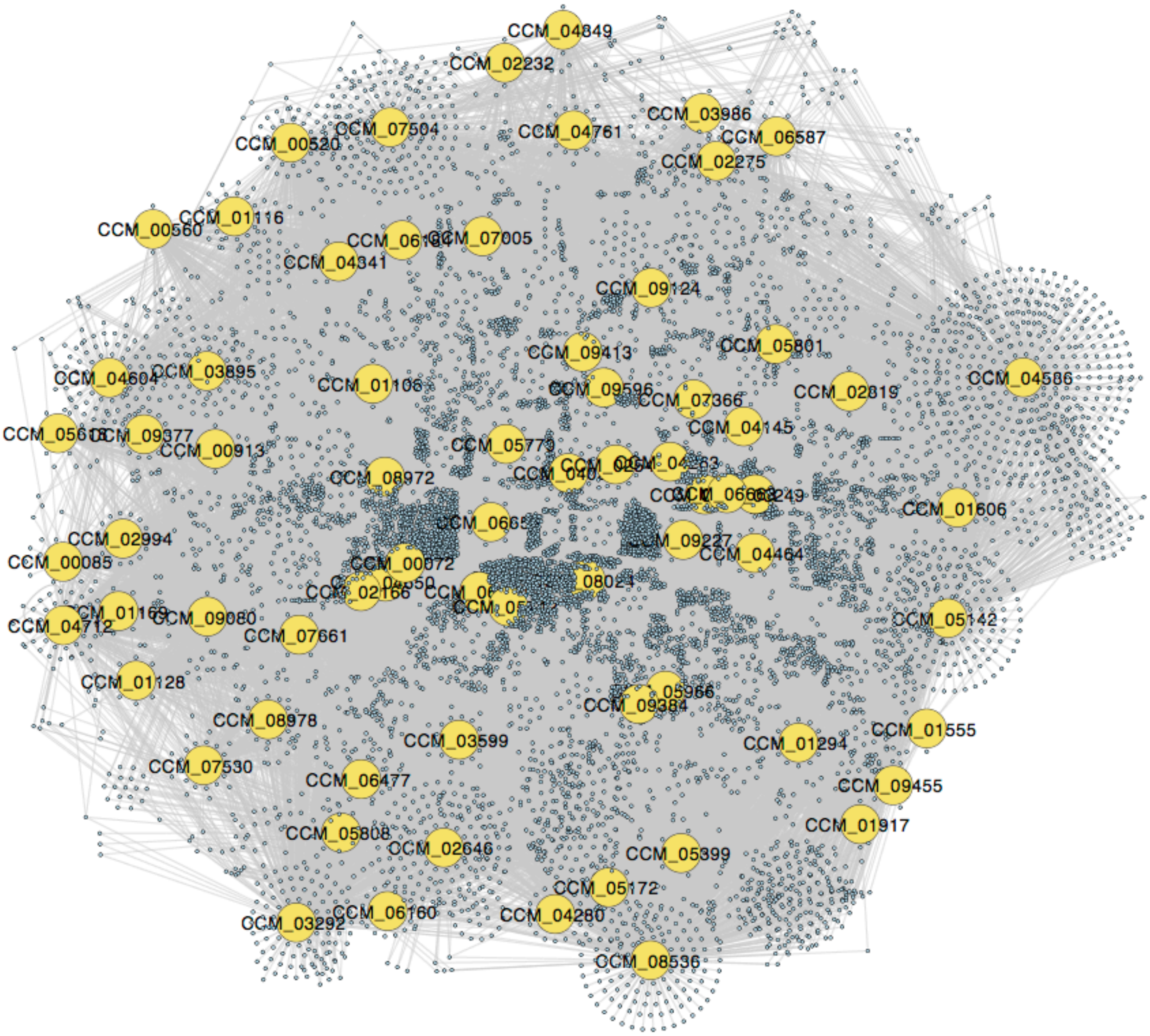

Figure S1. The genome-scale GRN. Large yellow and small blue nodes represent the transcription factors (TFs) and other genes, respectively. Grey lines represent the interactions between TFs and targets.
